# Diagnostic Performance of Serum Biomarkers Fibroblast Growth Factor 21, Galectin-3 and Copeptin for Heart Failure with Preserved Ejection Fraction in a Sample of Patients with Type 2 Diabetes Mellitus

**DOI:** 10.3390/diagnostics11091577

**Published:** 2021-08-30

**Authors:** Raluca D. Ianoș, Călin Pop, Mihaela Iancu, Rodica Rahaian, Angela Cozma, Lucia M. Procopciuc

**Affiliations:** 1 Department of Cardiology, Iuliu Hațieganu University of Medicine and Pharmacy, 400001 Cluj-Napoca, Romania; ralu_yannosh@yahoo.com; 2 Department of Cardiology, Emergency County Hospital, 430031 Baia Mare, Romania; 3 Faculty of Medicine Arad, “Vasile Goldis” Western University, 310045 Arad, Romania; 4 Department of Medical Informatics and Biostatistics, Iuliu Hațieganu University of Medicine and Pharmacy, 400349 Cluj-Napoca, Romania; 5 Department of Immunology, Emergency County Hospital, 400006 Cluj-Napoca, Romania; rodicarahaian@gmail.com; 6 Department of Internal Medicine, Iuliu Hațieganu University of Medicine and Pharmacy, 400015 Cluj-Napoca, Romania; angelacozma@yahoo.com; 7 Department of Medical Biochemistry, Iuliu Hațieganu University of Medicine and Pharmacy, 400349 Cluj-Napoca, Romania; luciamariaprocopciuc@yahoo.com

**Keywords:** biomarkers, heart failure, preserved ejection fraction, type 2 diabetes mellitus

## Abstract

More than half of the patients with heart failure have preserved ejection fraction (HFpEF), however evidence shows a mortality rate comparable to those with reduced ejection fraction. The aim of this study was to evaluate whether FGF21, galectin-3 and copeptin can be used as biomarkers to identify HFpEF in patients with confirmed type 2 diabetes mellitus (DM). Sixty-nine diabetic patients were enrolled and divided into two groups: patients with HFpEF (*n* = 40) and those without HFpEF (*n* = 29). The ability of the studied biomarkers to discriminate HFpEF cases from non-HFpEF subjects were evaluated by the area under the Receiver Operating Characteristics (ROC) curve and the 95% confidence interval (CI). Compared to patients without heart failure, those with HFpEF had significantly higher levels of FGF21 (mean 146.79 pg/mL vs. 298.98 pg/mL). The AUC value of FGF21 was 0.88, 95% CI: [0.80, 0.96], Se = 85% [70.2, 94.3], Sp = 79.3% [60.3, 92.0], at an optimal cut-off value of 217.40 pg/mL. There was no statistical significance associated with galectin-3 and copeptin between patient cohorts. In conclusion, galectin-3 and copeptin levels were not effective for detecting HFpEF, while FGF21 is a promising biomarker for diagnosing HFpEF in DM patients.

## 1. Introduction

Diabetes mellitus (DM) is an important risk factor for cardiovascular disease, representing a frequent cause of microvascular and macrovascular complications, cardiac damage including ischemic coronary artery disease diabetic cardiomyopa-thy, heart failure (HF) and autonomic neuropathy, with a cardiovascular mortality rate twice that of non-diabetic patients [1]. Mortality due to cardiomyopathy on account of DM is relatively high, diabetic cardiomyopathy (DCM) being evident in 60% of type 2 diabetic cases. It has been estimated that the prevalence of DM worldwide will increase from 2.8% in 2000 to 4.4% in 2030 [2].

Among patients with signs and symptoms of HF, approximately 50% have preserved left ventricular ejection fraction (HFpEF) [3]. Multiple comorbidities are common in both types of HF (preserved and reduced EF), but slightly more severe in HFpEF, in which approximately half of the patients have at least five major comorbidities [3]. In patients with HFpEF, a high proportion of deaths are due to cardiovascular events, but the proportion of non-cardiovascular deaths is higher in HFpEF than HFrEF. HFpEF incidence has increased from 47.8% to 52.3% between 2000 and 2010. The majority of observational studies have shown a similar risk of mortality in HFpEF compared to HFrEF, suggesting the importance of this pathology [3]. HFpEF develops from a combination of risk factors and comorbidities, including advanced age, female gender, obesity, systemic arterial hypertension and DM [4]. According to the European Heart Failure Management Guidelines, no treatment has so far been shown to reduce morbidity and mortality in HFpEF or intermediate ejection fraction [5,6]. The metabolic disturbances present in diabetic patients are often accompanied by cardiac changes consisting of local inflammation, oxidative stress, myocardial fibrosis and cardiomyocyte apoptosis. It is of great interest and utility to discover specific biomarkers that integrate these processes, in order to detect DCM at an early stage and evaluate their potential role in the introduction of targeted therapies to prevent progression of the disease to severe HF [7].

B-type natriuretic peptides (BNP and NT-proBNP) are diagnostic and prognostic biomarkers for congestive HF [6]. However, there are studies which indicate a limitation in the use of NT-proBNP for diagnosing HFpEF [8,9,10]. Therefore, new biomarkers and diagnostic strategies are needed to detect HFpEF.

Fibroblast growth factor 21 (FGF21), galectin-3 and copeptin [2,11] have been proposed as biomarker candidates to detect the early stages of cardiomyopathy.

In the literature, the role of galectin-3 (a beta-galactosidase binding lectin) is emphasized in the process of fibrosis, inflammation and cardiac remodeling [7]. At the cardiac level, its expression is low, but during cardiac injury, it is rapidly induced [7]. Although intracellular levels of galectin-3 correlate with tissue repair, its uncontrolled expression contributes to sustained activation of macrophages and myo-fibroblasts by dependent and independent transforming growth factor beta (TGF-β) pathways resulting in tissue fibrosis [7]. Galectin-3 is locally secreted by activated fibroblasts and macrophages, exerting its pro-fibrotic function by augmenting pro-liferation of myofibroblasts, extracellular matrix accumulation, macrophage infiltration and cardiac hypertrophy by stimulating TGF-β signaling pathways [7]. Plasma levels of galectin-3 have been proposed as a good biomarker for prediction and prognosis of left ventricular systolic dysfunction and HF in diabetic patients. Galectin-3 may be of therapeutic interest, since its inhibition prevents pro-inflammatory and pro-fibrotic mechanisms [7,12].

It is well known that hyperglycemia initiates DCM and contributes to various pathological processes of this disease. FGF21 is a polypeptide that plays a role in regulating glucose homeostasis and lipid metabolism, by reducing plasma glucose levels and lowering triglyceride levels in the liver and serum [13]. Recent clinical and subclinical studies have found that increased serum FGF21 is closely associated with DCM [14] and can be considered a potential biomarker. There is evidence that myocytes secrete FGF21 as an autocrine factor, to protect the heart from adverse cardiac remodeling [7,15]. However, whether an increased level of serum FGF21 is the basis of DCM pathogenesis or a key molecule involved in repairing the damage from DCM is so far unclear [13]. This biomarker has significant positive correlation with hypertension, DM, severity of HF, ischemic coronary artery disease and peripheral arterial disease [15,16,17]. There is indirect evidence that FGF21 is involved in cardiac remodeling and can be a therapeutic target in both metabolic and cardiovascular diseases [15].

Vasopressin secreted by the posterior pituitary gland is involved in osmoregulation with an important role in the pathophysiology of cardiac failure. In HF, the arginine vasopressin system contributes to the progression of left ventricular dysfunction by directly stimulating left ventricular hypertrophy and myocardial remodeling. Since vasopressin is unstable in plasma and has a low half-life, copeptin (C-terminal portion of pro-arginine vasopressin) was introduced as a vasopressin surrogate marker [18,19] and was investigated across the spectrum of cardiovascular diseases (including HF) as a prognostic marker [18]. A high copeptin level is independently associated with the incidence of DM, albuminuria and abdominal obesity [20,21,22].

The objectives of the present study were: (i) to evaluate whether FGF21 (an adiponectin with a role in regulating glucose and lipid homeostasis), copeptin (a precursor of pro- arginine vasopressin and a marker of neurohormonal activation) and galectin-3 (a marker of inflammation and fibrosis) can be used as biomarkers for the diagnosis of HFpEF in patients with type 2 DM; (ii) to determine the levels of biomarkers in patients with type 2 DM; and (iii) to assess the correlations between these biomarkers and echocardiographic parameters in the same population

## 2. Materials and Methods

### 2.1. Study Design and Characteristics

Between February 2019 and November 2020, 86 patients with DM were evaluated for inclusion in the study at the Nicolae Stăncioiu Heart Institute Cluj-Napoca, Romania, and at the Dr Constantin Opriș Emergency County Hospital, Baia Mare, Romania.

Institutional Review Board Statement: The study was conducted according to the guidelines of the Declaration of Helsinki, and approved by the Ethics Committee of Iuliu Hatieganu University of Medicine and Pharmacy, Cluj-Napoca, Romania (protocol code 48/11 March 2019) and Emergency County Hospital Baia Mare, Romania (no. 6361/28 February 2020). An informed consent was obtained from all subjects involved in the study.

The inclusion criteria consisted of the presence of DM under treatment, a left ventricular (LV) ejection fraction (EF) ≥ 50% and age over 18 years old. The exclusion criteria were recent hospitalization due to HF decompensation for patients with HF, severe valvular disease, hypertrophic or infiltrative cardiomyopathy, congenital heart disease, chronic obstructive pulmonary disease stage GOLD 3 or 4, pericardial disease, atrial fibrillation with high ventricular response > 100 bpm, severe renal failure (eGFR by CKD-EPI < 30 mL/min/1.73 m^2^), severe anemia (hemoglobin < 7 g/dL) and neoplasia. After excluding subjects with intermediate LVEF (between 40–49%), we enrolled in our study 69 diabetic patients who fulfilled the inclusion criteria and we divided them into two groups: patients fulfilling the criteria for a diagnosis of HFpEF and patients without HF. We defined HFpEF according to a consensus recommendation from the Heart Failure Association of the European Society of Cardiology, using the HFA-PEFF diagnosis algorithm [4] based on clinical assessment revealing signs and symptoms of HF [4,23], echocardiographic measurements of function and morphology [4,24,25,26,27] and the level of natriuretic peptides. A HFA-PEFF score ≥ 5 points defines HFpEF. The score includes three domains (morphological, functional and biomarker) with major (2 points) and minor criteria (1point). The major criteria include septal e’ < 7 cm/s, or lateral e’ < 10 cm/s, or average E/e’ ≥ 15 or TR velocity > 2.8 m/s (PASP > 35 mmHg), left atrial volume index > 34 mL/m^2^ or left ventricular mass index ≥ 149 g/m^2^ in men or ≥122 g/m^2^ in women and relative wall thickness > 0.42, NT-proBNP > 220 pg/mL in sinus rhythm or >660 pg/mL in atrial fibrillation. The minor criteria are average E/e’ 9–14 or GLS < 16%, left atrial volume index 29–34 mL/m^2^ or relative wall thickness > 0.42, NT-proBNP 125–220 pg/mL in sinus rhythm or 365–660 pg/mL in atrial fibrillation.

### 2.2. Biomarkers

At the time of admission, blood samples were collected in serum separator tubes with clot activator (BD Vacutainer CAT) and allowed to clot at room temperature for no more than 2 h before centrifugation for 15 min at 1000× *g* to obtain serum. The samples were separated into Eppendorf tubes and stored at −80 °C until analysis. The NT-proBNP analysis was performed by an electrochemiluminescent immunoassay (Roche Diagnostics, Mannheim, Germany). We evaluated the seric concentration of the biomarkers through quantitative enzyme-linked immunosorbent assay (ELISA): FGF21(Catalog No E-EL-H0074, sensitivity 18.75 pg/mL, detection range: 31.25–2000 pg/mL, ElabscienceBiotechnology Inc., Houston, TX, USA), Galectin-3 (Catalog No E-El-H1470, sensitivity 0.1 ng/mL, detection range 0.16–10 ng/mL, Elascience Biotechnology Inc., Houston, TX, USA), Copeptin (Catalog No E-EL-H0851, sensitivity 18.75 pg/mL, detectionrange 31.25–2000 pg/mL, Elabscience Biotechnology Inc., Houston, TX, USA).

### 2.3. Echocardiographic Assessment

All subjects underwent a complete two-dimensional transthoracic echocardi-ography (Philips CX 50, xMATRIX, Philips Ultrasound Inc., Bothell, WA, USA) examination prior to inclusion in the study protocol, using the cardiology software application. LVEF was obtained by using Simpson’s method, with LV end-diastolic (EDV) and end-systolic volumes (ESV) acquired in the apical 4-chamber. Transmitral inflow, the peak velocities of early filling (E) and the deceleration time of E were evaluated by pulsed wave blood flow Doppler at the mitral valve leaflet tips in apical 4-chamber view. The early peak diastolic mitral annulus velocity (e’) was obtained by pulsed wave tissue Doppler in the LV septum and lateral wall. Pulmonary arterial systolic pressure was calculated using the modified Bernoulli equation as 4 x peak tricuspid regurgitation (TR) velocity adding the estimated right atrial pressure.

### 2.4. Statistical Analysis

Clinical features, laboratory and echocardiographic data were described as arithmetic mean (standard deviation), or as counts (percentage) for categorical variables. Patients were divided into two groups according to the presence or absence of HFpEF. The continuous variables were checked for univariate normal distribution through kurtosis-skewness analysis and Anderson-Darling test. The serum biomarkers FGF21, NT-proBNP and copeptin were consistent with log-normal distributions and were modeled using a natural logarithmic transformation. The log-transformed data were reported using geometric mean as a measure of central tendency and geometric standard deviation as a measure of log-normal dispersion. The comparisons between the studied groups concerning the serum biomarker values were performed based on their log-transformed data using Student-t test with equal variances or Welch’s *t*-test. For continuous variables with deviations from normal distribution, we used the Mann-Whitney U test for comparisons between the studied groups. The associations between nominal variables and HF in patients with type 2 DM were tested using Chi-square or Fisher’s exact test. Pearson or Spearman rank correlation coefficients were used to assess the correlation between the log-transformed biomarker values and clinical, laboratory and echocardiographic variables. The Pearson correlation coefficient was used to quantify the strength of a linear correlation between two quantitative variables assuming that each of the variables followed a normal distribution; otherwise, the Spearman coefficient was used.

The ability of biomarkers FGF21, copeptin and galectin-3 to distinguish HFpEF cases from non-HFpEF subjects was evaluated by the area under the Receiver Operating Characteristics (ROC) curve and the 95% confidence interval (CI), percentage of correct predictions (PCP) with their 95% CI, sensitivity and specificity (95% CI). A PCP value close to 1 denoted that the tested model had a good discrimination for HF The optimum cut-off point was obtained using the Youden index. All inferential analyses were performed using two-sided tests with statistical significance set at an estimated *p*-value < 0.05. All statistical analyses were performed in R software version 4.0.4 (R Foundation for Statistical Computing, Vienna, Austria).

## 3. Results

### 3.1. Baseline Characteristics of the Sample of Patients with Type 2 DM

Eighty-six consecutively selected patients were evaluated for inclusion in the study. Considering the inclusion and exclusion criteria, the studied sample consisted of 69 patients with type 2 DM divided into 2 groups: patients with HFpEF (*n* = 40, LVEF ≥ 50%) and those without HFpEF (*n* = 29). The distributions of baseline characteristics in each group are described in Table 1.

Patients with HFpEF had significantly different HbA1c values than patients without HF (*p* = 0.008). Concerning NYHA classification, we noticed that stages II and III were more frequent in patients with HFpEF (90% of cases). The presence of multivessel coronary artery disease was more frequent in patients with HFpEF compared to patients without HF (*p* = 0.031).

### 3.2. Comparisons of Serum FGF21, Galectin-3, Copeptin and Echocardiographic Parameters in Patients with Type 2 Diabetes Mellitus Grouped by Heart Failure

The NT-proBNP level was significantly higher in DM patients with HFpEF compared to patients without HEpEF (1279.83 ± 3.08 vs. 153.26 ± 2.75, *p* < 0.001). Similarly, there were significant differences between the two groups regarding the FGF21 level (*p* < 0.001). There was no statistical difference in galectin-3 and copeptin concentrations between the two studied groups (Table 2). Echocardiographic parameters such as LVEF (*p* = 0.003), LVESV, *p* = 0.014) and E/e, left atrial (LA) surface, TR velocity (*p* < 0.001) were significantly different between DM patients with HFpEF and those without HFpEF.

### 3.3. Evaluation of FGF21, Copeptin and Gal-3 as Markers of HFpEF in Patients with Type 2 DM

Binomial logistic regression was performed to evaluate the association betweenFGF21, copeptin and galectin-3 and the odds of HFpEF (Table 3). In the univariable regression analysis, there was no statistical evidence of a significant association of galectin-3 and copeptin with the odds of HFpEF in patients with type 2 DM (*p* = 0.240 for galectin-3 and *p* = 0.628 for copeptin), but the biomarker FGF21 (*p* = 0.001) was significantly associated with the odds of HFpEF.

**Figure 1:**
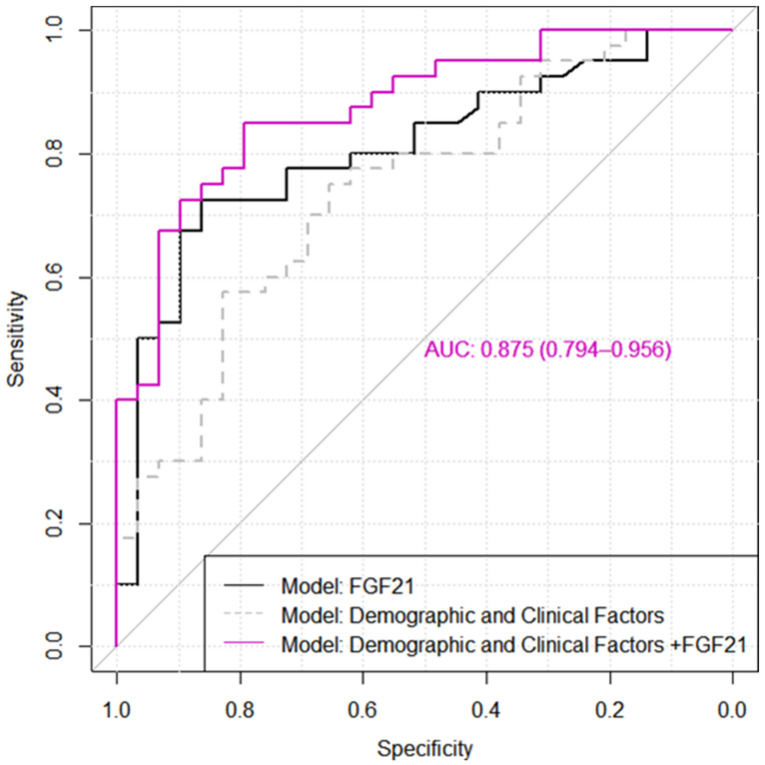
The ROC curves for distinction between type 2 diabetes mellitus patients with heart failure with preserved ejection fraction from those without heart failure. The demographic and clinical factors included in the tested model were age, gender, BMI, DBP, HbA1c and previous MI (See Table 3).

At the FGF21 optimal cut-off value of 217.40 pg/mL, 29 out of 40 HFpEF patients were identified as patients with HF. The ROC curve of FGF21 is presented in Figure 1 with an AUC value of 0.81, 95% CI: [0.70, 0.91]. After adjusting for demographic and clinical covariates such as age, gender, body mass index, diastolic blood pressure, HbA1c and previous myocardial infarction, the biomarker FGF21 remained in a significant association with the odds of HF (*p* = 0.001). The explanatory multivariable model demonstrated a good distinction between HFpEF and non-HFpEF patients with type 2 DM (AUC = 0.88, 95% CI: [0.80, 0.96], PCP = 81.16%, 95% PCP: [71.93, 90.39], Se = 85% [70.2, 94.3], Sp = 79.3% [60.3, 92.0].

Although the addition of covariates (demographic and clinical factors) provided a greater model ability to discriminate between HFpEF and non-HFpEF patients with type 2 DM, the results showed only a tendency towards statistical significance: DeLong’s test: ΔAUC = 0.07, *p* = 0.109 (Figure 1).

### 3.4. Correlations among FGF21, Galectin-3, and Copeptin with Clinical, Laboratory Data and Echocardiographic Variables in All Samples of Patients with Type 2 DM

In bivariate correlation analysis between the studied biomarkers and echocardiographic characteristics (Table 4), there was a significant positive correlation of FGF21 with LVEDV (Spearman’s correlation coefficient, ρ = 0.32, *p* = 0.008), LVESV (Pearson’s correlation coefficient, r = 0.39, *p* < 0.001), LA surface (Pearson’s correlation coefficient, r = 0.26, *p* = 0.028) and LA volume (Spearman’s correlation coefficient, ρ = 0.29, *p* = 0.017).

Copeptin had a negative correlation with LVEDV (ρ = −0.30, *p* = 0.014) and LSESV (r = −0.31, *p* = 0.008), and a linear positive correlation with E wave velocity (r = 0.25, *p* = 0.036). Galectin-3 was positively correlated only with LA volume index, TR velocity and pulmonary artery systolic pressure (Table 4).

## 4. Discussion

Patients with HFpEF represent nearly 50% of the HF population and is associated with a decreased life expectancy [26,28]. This impaired prognosis, however, is not fully explained by the associated comorbidities such as hypertension, diabetes mellitus, atrial fibrillation and obesity, as higher mortality rates have been reported in patients with HFpEF than in those with similar comorbidities but without HF [28]. Regarding NT-ProBNP, which has a high specificity but a lower sensitivity for the diagnosis of HFpEF, new biomarkers need to be investigated.

In this study, we demonstrated the utility of FGF21 in diagnosing HFpEF in diabetic patients, with a high sensitivity and specificity at a cut-off value of 217.4 pg/mL. FGF21 had a significant association with the odds of HF (*p* = 0.001), even after adjusting for demographic and studied clinical covariates. FGF21, in combination with demographic and clinical factors, had a better ability to distinguish between diabetic HFpEF and non-HF patients, but the results were not statistically significant, only showing a trend towards significance, possibly due to the small number of patients included.

These results are in accordance with the first study conducted by Ruey-Hsing in 2016 [6], which demonstrated the association between elevated levels of FGF21 and diastolic dysfunction in adult humans.

In recent years, there has been a lot of evidence to suggest that that increased serum FGF21 levels are found in obese, insulin-resistant patients, and those with metabolic syndrome [29]. Additionally, serum FGF21 levels were higher in diabetic patients with low urinary glucose excretion compared to those with high urinary glucose excretion (pg/mL, 429.4 vs. 263.5, *p* = 0.002) [30]. Elevated levels of FGF21 in serum during the early stages of various metabolic diseases are considered a compensatory response by the organism. Therefore, FGF21 is regarded as a hormone in response to stress and an early diagnostic marker of disease [13]. The results in our study also demonstrated a significant correlation between FGF21 levels and obesity, BMI, body surface area, total cholesterol and LDL cholesterol.

FGF21 binds to the FGF receptor (FGFR) in the presence of the co-factor ß-Klotho, acts as an adipokine in order to determine glucose uptake in adipocytes and further, increases insulin sensitivity [13,31,32]. In a recent review, the protective role of FGF21 was highlighted, especially in pathological processes such as suppressing apoptosis in the myocardium, reducing inflammation in cardiomyocytes and oxidative stress, and promoting fatty acid oxidation [13]. It is suggested that DCM can be delayed through the application of injectable exogenous FGF21, providing possible therapeutic targets in this disease [13]. Studies performed on mice suggest that exogenous FGF21 protects people from heart injury in DCM by antagonizing oxidative stress, but the quantity of exogenous FGF21 administered in these studies was considerably higher than that in normal physiological conditions [13,33]. Expression of FGF21 in the heart is under the control of the protein deacetylase Sirt1 (sirtuin1). In an environment with high levels of sugar and fat, activation of the Sirt1 pathway generates cardiac secretion of FGF21, which acts in an autocrine manner to prevent oxidative stress in cardiomyocytes, by promoting the expression of certain antioxidant genes (Ucp2, Ucp3, or Sod2) [34]. In conclusion, FGF21 performs a role in suppressing apoptosis in myocardial cells induced by oxidative damage both in vitro and in vivo, protection occurring through modulation of apoptosis-related genes and the oxidoreductase system. However, these findings are based on animal studies, with a lack of research data from clinical studies, requiring further investigations [13].

The diagnostic value of galectin-3, a biomarker of myocardial fibrosis and inflammation, has not been extensively studied. Van Kimmenade et al. found that galectin-3 values were significantly higher in subjects with HF, compared to those without HF. At a cut-off value of 6.88 ng/mL, galectin-3 had a sensitivity of 80% and a specificity of 52% in the diagnosis of HF as the etiology of dyspnea, with a receiver operating characteristic [ROC] curve analysis of 0.72 [35].

Another study conducted by Trippel et al. in a population with cardiovascular risk factors, found that at a cut-off value of 13.57 ng/mL, galectin-3 had a sensitivity of 61% and a specificity of 73% for diagnosis of HFpEF. The AUC of Gal-3 was 0.71. Furthermore, patients with galectin-3 ≥ 13.4 ng/mL developed incident HFpEF significantly more often than patients with galectin-3 < 13.4 ng/mL [36].

In the COACH study (de Boer et al.), higher levels of galectin-3 were associated with higher rates of re-hospitalization and death in HFpEF, but not in patients with HFrEF [37].

A systematic review and meta-analysis [24] which investigated the diagnostic potential of biomarkers in HFpEF found that the studies investigating galectin-3 as a diagnostic biomarker of HFpEF were conducted in Asia, with cut-off values of 9.55 ng/mL, 17.8 ng/mL, 20.12 ng/mL [28,38,39,40].

The study conducted by Salman et al. revealed that galectin-3 levels were 15.1 ng/mL in patients with type 2 DM and macroalbuminuria, 8.3 ng/mL in patients with type 2 DM and normoalbuminuria, and 9.8 ng/mL in patients with type 2 DM and normoalbuminuria, the results being statistically significant (*p* = 0.01 and *p* = 0.05, respectively). They also found a negative correlation between the galectin-3 level and eGFR [41].

A Chinese study on 284 diabetic patients (Jin Qi-hui et al.) demonstrated that the mean galectin-3 level was 27.4 ng/mL in patients with DM. At a serum galectin level > 25 ng/mL, they demonstrated that galectin-3 was a risk factor for HF [42].

The study conducted by Edelman et al. revealed that galectin-3 was associated with HFpEF and fibrosis [43]. Pecherina et al. investigated the serum galectin-3 levels in patients with ST-segment elevation myocardial infarction (STEMI) and preserved LVEF (HFpEF) compared to those with HFrEF, and found that this biomarker correlated with the parameters reflecting diastolic dysfunction in patients with HFpEF, and with LVEF and left ventricular end-systolic volume/diameter in patients with HFrEF [44].The TOPCAT trial investigated the biomarker profiles of patients with diabetes and HFpEF compared to patients without DM. The authors reported levels of 22.0 ng/mL for galectin-3 in patients with DM with HFpEF, higher than in patients without diabetes, 20.0 ng/mL [45].

In our study, there was no statistically significant difference in galectin-3 concentrations between the studied groups; thus, we did not find a possible association with the risk of HF. The mean value of galectin-3 in our studied sample of diabetic patients was 11.9 ng/mL, lower than the value obtained by Jin Qi-hui et al. [42] in a Chinese population, and also lower than the value reported by the TOPCAT trial [45]. To our knowledge, studies enrolling Caucasian subjects with type 2 DM for evaluating the diagnostic performance of galectin in HFpEF are lacking. Schill et al. analyzed copeptin levels in 5297 individuals without prevalent HF from the Malmö Preventive Project and found that in older adults this biomarker could predict the development of HF [46]. Noor et al. published their results regarding the relationship of copeptin with DM progressing towards diabetic nephropathy and found significantly higher copeptin levels in subjects with a positive family history of DM compared to those with no history of DM (pg/mL, 243.77 vs. 165.2, *p* = 0.025). The same study found that the level of copeptin was negatively correlated with eGFR and there was no correlation between the level of copeptin and the level of HbA1c [47].

The copeptin level is higher and has a prognostic value in patients with HFrEF, but its role in HFpEF is still underexplored [11,48]. In acute HF, copeptin has been shown to be a prognostic predictor of HF hospitalization and mortality [49,50]. In a report from the prospective KaRen study, Hage C. et al. found that copeptin levels did not differ between patients with and without diastolic dysfunction [11]. Our results confirmed those of Hage C. et al.

FGF21 significantly correlated with echocardiographic parameters such as the left atrial volume, left atrial surface and pulmonary artery systolic pressure and had a borderline correlation with E/e’ ratio, with results comparable to those obtained by Ruey-Hsing et al. in 2016 [6]. On the other hand, we did not find an association between the other biomarkers (galectin-3 and copeptin) and echocardiographic measurements of diastolic dysfunction.

In the present study, there was a significant difference in HbA1c levels among diabetic patients with and without HFpEF. The levels of HbA1c were significantly higher in diabetic patients with HFpEF compared to diabetic patients without HFpEF (*p* = 0.008). In our study, multivariate analysis confirmed that Hb1Ac is an independent risk factor for HF in diabetic patients (*p* = 0.025). Other studies have evaluated the association between Hb1Ac levels and the risk of HF in patients with diabetes. The study performed by Pazin Filho found that HbA1c is an independent risk factor for HF in patients with diabetes with or without cardiovascular diseases [51] and Zhao confirmed these results in African American and white patients with diabetes [52].

Moreover, Lind suggested that the risk of hospitalization increased in diabetic patients with poor glycemic control (HbA1c) > 7%) [53], and Erqou revealed that a higher HbA1c level was associated with a significantly increased risk for congestive heart failure [54]. Poor glycemic control is associated with a 1.56-fold increased risk of HF among adult patients with diabetes according to the results presented by Iribarren [55].

As far as we know, this is the first study conducted in a Caucasian population to evaluate for HFpEF in diabetic patients the diagnostic performance of three specific biomarkers that are involved in different types of pathological mechanisms of HF (FGF21 regulates glucose and lipid homeostasis, copeptin is involved in neurohormonal activation and galectin-3 promotes inflammation and fibrosis). FGF21 is a stress inducible hormone that plays important roles in regulating energy balance and glucose and lipid homeostasis [56] and there are studies demonstrating that myocytes secrete FGF21 as an autocrine factor, in order to protect the heart from adverse cardiac remodeling [7,15]. Studies conducted on rodents or on primates demonstrated that the administration of FGF21 brings considerable pharmacological benefits on a cluster of obesity-related metabolic complications, including a reduction in fat mass and alleviation of hyperglycemia, insulin resistance, dyslipidemia, cardiovascular disorders and non- alcoholic steatohepatitis (NASH).

Because native FGF21 is not suitable for clinical use due to its poor pharmacokinetic and biophysical properties, there has developed a large number of long-acting FGF21 analogues–ß-and agonistic monoclonal antibodies for theFGFR1–ß-klotho receptor complexes [56]. The clinical trials including patients with obesity, DM type 2 and nonalcoholic steatohepatitis, demonstrated substantial improvements in dyslipidemia, hepatic fat fractions and serum markers of liver fibrosis, whereas the primary end points of glycemic control have not been met [56]. In this context, several current drugs used for treatment inDM type 2 are highly effective for glucose lowering, but lack the considerable therapeutic effects of FGF21 on dyslipidemia. An association of FGF21- based pharmacotherapy with these glucose- lowering agents to treat multiple obesity- related metabolic complications might constitute a promising strategy that worth further exploration [56]. In our study we showed that FGF21 can represent a novel candidate biomarker for diagnosis in HFpEF in diabetic patients, facilitating its early detection, but further studies are needed to confirm our finding, with a larger number of patients. Furthermore, the utility of this biomarker is enhanced by the fact that FGF21 could be used in the future as a target therapy in metabolic and cardiovascular disease.

The main limitation of the present study was the relatively small sample size, which also restricted the testing of a multivariable model containing other known covariates such as coronary artery disease or NYHA class. The biomarker levels were measured only at the time of admission, and we do not have data about the change in their concentration at the time of readmission. Further assessment of these biomarkers (FGF21, galectin-3, copeptin) for HF diagnosis in patients with HFpEF should be conducted with a larger study cohort.

## 5. Conclusions

Neither galectin-3 nor copeptin showed promise as a biomarker in diagnosing HFpEF. In contrast with the negative findings related to galectin-3 and copeptin, fibroblast growth factor 21 may be a novel candidate biomarker for the diagnosis of HFpEF in diabetic patients. Further studies are needed with a larger sample of patients.

## Figures and Tables

**Table 1:** Demographic, clinical and para-clinical characteristics of patients with type 2 DM.

	All DM Samples (*n* = 69)	DM without HFpEF (*n*_1_ = 29)	DM with HFpEF (*n*_2_ = 40)	*p*-Value
Age, years ^(a)^	64.65 ± 9.83	63.83 ± 7.70	65.25 ± 11.17	0.557
Male ^(b)^	37 (53.62)	18 (62.07)	19 (47.50)	0.231
Body mass index, kg/m^2^ ^(a)^	32.43 ± 6.69	30.94 ± 7.25	33.50 ± 6.11	0.117
Body Surface Area ^(a)^	1.99 ± 0.21	2.00 ± 0.24	1.99 ± 0.18	0.866
SBP, mmHg ^(a)^	138.40 ± 19.68	136.60 ± 18.57	139.70 ± 20.59	0.519
DBP, mmHg ^(a)^	79.67 ± 10.96	77.45 ± 10.20	81.28 ± 11.33	0.153
Smoking ^(b)^	15 (21.74)	8 (27.59)	7 (17.50)	0.316
Medical history ^(b)^				
Hypertension	60 (86.96)	27 (93.10)	33 (82.50)	0.285
Atrial fibrillation	9 (13.04)	1 (3.45)	8 (20.00)	0.069
Paroxysmal atrial fibrillation	3 (4.35)	0 (0.00)	3 (7.50)	0.258
Previous stroke	2 (2.90)	0 (0.00)	2 (5.00)	0.506
Previous MI	32 (46.38)	10 (34.48)	22 (55.00)	0.092
COPD	3 (5.80)	1 (3.45)	3 (7.69)	0.631
PAD	5 (7.25)	5 (17.86)	0 (0.0)	0.009
Medication ^(b)^				0.765
Insulin	30 (43.48)	12 (41.38)	18 (45.00)	
Oral antidiabetic drugs ^(b)^	39 (56.52)	17 (58.62)	22 (55.00)	
Laboratory data ^(a)^				
HbA1C, %	7.65 [7.20, 9.25]	7.42 [6.90, 7.69]	8.84 [7.30, 9.96]	0.008 *
Fasting blood glucose, mg/dL	187.50 ± 74.12	171.50 ± 58.59	199.10 ± 82.38	0.128
Total cholesterol, mg/dL	166.60 ± 46.16	156.60 ± 41.67	173.80 ± 48.37	0.127
LDL cholesterol, mg/dL	93.66 ± 38.42	88.13 ± 38.97	97.66 ± 38.01	0.313
HDL cholesterol, mg/dL	38.74 ± 9.19	37.32 ± 8.44	39.77 ± 9.68	0.277
Triglycerides, mg/dL	155.20 [112.40, 204.00]	156.40 [124.00, 181.10]	150.50 [107.20, 224.00]	0.918
eGFR, mL/min/1.73 m^2^ by CKD-EPI	73.69 ± 24.49	78.07 ± 21.70	70.53 ± 26.14	0.209
Hemoglobin, g/dL	13.38 ± 1.71	13.73 ± 1.76	13.13 ± 1.65	0.152
Uric acid mg/dL	6.36 ± 1.88	5.92 ± 1.06	6.68 ± 2.26	0.070
Coronary angiography #, ^(b)^				0.031 *
Absent	4 (5.80)	4 (16.00)	0 (0.00)	
Monovessel CAD	15 (21.74)	4 (16.00)	11 (32.35)	
Multivessel CAD	40 (57.97)	17 (68.00)	23 (67.65)	

^(a)^ Data are presented as arithmetic mean ± sample standard deviation or median [25th percentile; 75th percentile], ^(b)^ absolute frequencies (relative frequencies, %). *p*-values were obtained by Student-*t* test with equal variances, Welch’s *t*-test, Chi-square test or Fisher’s exact test, * significant results (*p* < 0.05); # complete case data *n* = 59; HFpEF = heart failure with preserved ejection fraction, SBP = systolic blood pressure, DBP = diastolic blood pressure, NYHA = New York Heart Association, MI = myocardial infarction, COPD = chronic obstructive pulmonary disease, PAD = peripheral arterial disease, HbA1c = glycated hemoglobin, eGFR = estimated glomerular filtration rate; CKD-EPI = Chronic Kidney Disease Epidemiology Collaboration, CAD = coronary artery disease.

**Table 2:** Comparison of FGF21, galectin-3, copeptin and echocardiographic parameters between the studied groups.

	All DM Samples (*n* = 69)	DM without HFpEF (*n*_1_ = 29)	DM with HFpEF (*n*_2_ = 40)	*p*-Value
FGF21, pg/mL ^(a)^	221.72 ± 2.01	146.79 ± 1.76	298.98 ± 1.90	<0.001 *^,(d)^
Gal-3, ng/mL ^(b)^	11.91 ± 1.52	11.66 ± 1.71	12.10 ± 1.36	0.239
Copeptin, pg/mL ^(a)^	321.04 ± 2.55	348.35 ± 2.84	302.59 ± 2.36	0.541 ^(d)^
LV ejection fraction, % ^(b)^	54 [51, 59]	57 [54, 61]	53 [50, 55]	0.001 *
LV EDV, ml ^(c)^	108 [90, 126]	103 [89, 112]	116 [94.25, 138.20]	0.061
LV ESV, ml ^(b)^	50.4 ± 20.6	43.4 ± 17.2	55.5 ± 21.6	0.014 *
LVDd, mm ^(b)^	49.8 ± 5.1	50.3 ± 4.6	49.5 ± 5.5	0.539
LVSd, mm ^(b)^	29.0 ± 6.4	28.3 ± 6.2	29.6 ± 6.6	0.425
IVST, mm ^(b)^	12.3 ± 1.7	12.2 ± 1.7	12.3 ± 1.6	0.886
PWT, mm ^(b)^	12.0 ± 1.6	11.8 ± 1.8	12.1 ± 1.5	0.355
LA volume index, mL/m^2^ ^(c)^	30.0 [25.6, 35.0]	27.3 [24.0, 29.8]	32.31 [28.1, 41.3]	0.001 *
LA surface, cm^2^ ^(b)^	22.9 ± 6.8	19.3 ± 4.3	25.5 ± 7.2	<0.001 *
E wave velocity, m/s ^(b)^	0.8 ± 0.3	0.7 ± 0.2	0.9 ± 0.3	0.002 *
A wave velocity, m/s ^(b)^	0.8 ± 0.2	0.8 ± 0.2	0.9 ± 0.2	0.111
Mitral E/A ratio ^(b)^	0.9 ± 0.3	0.9 ± 0.4	0.9 ± 0.3	0.722
TDE, msec ^(b)^	217.8 ± 39.9	230.8 ± 33.5	208.4 ± 41.9	0.020 *
Septal e’ velocity, cm/s ^(b)^	7.2 ± 1.4	7.9 ± 1.3	6.7 ± 1.2	<0.001 *
Lateral e’ velocity, cm/s ^(b)^	8.6 ± 1.9	9.8 ± 1.5	7.7 ± 1.6	<0.001 *
E/e’ mean ratio ^(b)^	10.7 ± 3.9	8.2 ± 2.5	12.37 ± 3.9	<0.001 *
Relative wall thickness ^(b)^	0.48 ± 0.1	0.46 ± 0.06	0.49 ± 0.08	0.058
TR velocity, m/s ^(b)^	2.4 ± 0.6	2.06 ± 0.42	2.69 ± 0.61	<0.001 *
PASP, mmHg ^(b)^	30.6 ± 15.6	21.7 ± 7.9	37.1 ± 16.6	<0.001 *

Data are presented as ^(a)^ geometric mean ± geometric standard deviation or ^(b)^ arithmetic mean ± sample standard deviation or ^(c)^ median [25th percentile; 75th percentile], *p*-values were obtained from Student-t tests for independent groups or Mann-Whitney U test; ^(d)^ Student-*t* test applied on log-transformed data; * significant results (*p* < 0.05); DM = type 2 diabetes mellitus; LVEDV = left ventricular end-diastolic volume, LVESV = left ventricular end-systolic volume. LVEDP = left ventricular end-diastolic pressure, LA = left atrial, IVST interventricular septum thickness s, PWT = posterior wall thickness, LV = left ventricular, LVDd = left ventricular end-diastolic dimension, LVSd = left ventricular end-systolic dimension, TDE = deceleration time, TR velocity = tricuspid regurgitation peak velocity, PASP= pulmonary artery systolic pressure FGF21-fibroblast growth factor; Gal-3 = galectin-3; HFpEF = heart failure with preserved ejection fraction.

**Table 3:** Logistic regression analysis of factors used to separate patients with diabetes and heart failure from those without heart failure.

Variables	Univariable Model	*p*-Value	Multivariable Model	*p*-Value
	Crude OR [95% CI]		Adjusted OR [95% CI]	
FGF21 (pg/mL)	5.16 [2.19, 15.47]	0.001 *	8.80 [2.95, 36.30]	0.001 *
Age (years)	1.16 [0.71, 1.90]	0.551	2.52 [1.12; 6.68]	0.039 *
Gender ^(a)^	0.55 [0.20, 1.45]	0.233	0.67 [0.16, 2.72]	0.574
BMI (kg/m^2^)	1.50 [0.91, 2.55]	0.118	0.69 [0.31, 1.44]	0.340
DBP (mmHG)	1.45 [0.88, 2.50]	0.157	1.20 [0.64, 2.38]	0.573
HbA1c (%)	1.99 [1.14, 3.93]	0.0267 *	2.27 [1.19, 5.13]	0.025 *
Previous MI	2.32 [0.88, 6.40]	0.094	3.81 [0.99, 17.19]	0.061

FGF21 = fibroblast growth factor 21, BMI = body mass index, DBP = diastolic blood pressure, HbA1c = glycated hemoglobin; MI = myocardial infarction; * statistical significance: *p* < 0.05; Multivariable Model: FGF21 adjusted for demographic and clinical covariates; OR = odds ratio; ^(a)^ Reference category: Gender = F.

**Table 4:** Matrix of correlation coefficients (95% CI) between FGF21, galectin-3 and copeptin with echocardiographic variables.

Variables	Log FGF21	Log Copeptin	Gal-3
	Correlation Coefficient (95% CI)	Correlation Coefficient (95% CI)	Correlation Coefficient (95% CI)
LV EF, %	−0.26 [−0.47, 0.02] ^(b)^	0.20 [−0.05, 0.42] ^(b)^	−0.14 [−0.37, 0.11] ^(b)^
LV EDV, ml	0.32 [0.08, 0.52] ^(b),^*	−0.30 [−0.50, −0.06] ^(b),^*	0.21 [−0.03, 0.43] ^(b)^
LV ESV, ml	0.39 [0.17, 0.57] ^(a),^*	−0.31 [−0.51, −0.08] ^(^^a),^*	0.20 [−0.04, 0.42] ^(a)^
LA volume index, mL/m^2^	0.29 [0.05, 0.49] ^(b),^*	−0.09 [−0.33, 0.15] ^(b)^	0.25 [0.01, 0.47] ^(b),^*
LA surface, cm^2^	0.26 [0.03, 0.47] ^(a),^*	−0.05 [−0.29, 0.19] ^(a)^	0.12 [−0.12, 0.34] ^(a)^
E wave velocity, m/s	0.20 [−0.04, 0.41] ^(a)^	0.25 [0.02, 0.46] ^(^^a),*^	0.03 [−0.20, 0.27] ^(a)^
A wave velocity, m/s	−0.01 [−0.26, 0.24] ^(a)^	0.11 [−0.14, 0.35] ^(a)^	0.01 [−0.23, 0.26] ^(a)^
Mitral E/A ratio	0.19 [−0.06, 0.42] ^(a)^	0.25 [0.00, 0.47] ^(a)^	<0.01 [−0.25, 0.24] ^(a)^
TDE, msec	−0.16 [−0.38, 0.08] ^(a)^	−0.12 [−0.35, 0.12] ^(a)^	<0.01 [−0.23, 0.24] ^(a)^
Septal e’ velocity, cm/s	−0.19 [−0.41, 0.05] ^(a)^	−0.03 [−0.27, 0.21] ^(a)^	−0.07 [−0.30, 0.17] ^(a)^
Lateral e’ velocity, cm/s	−0.21 [−0.42, 0.03] ^(a)^	−0.01 [−0.25, 0.23] ^(a)^	0.03 [−0.21, 0.26] ^(a)^
E/e’ mean ratio	0.23 [−0.01, 0.44] ^(a)^	0.24 [0.00, 0.45] ^(a)^	0.06 [−0.18, 0.29] ^(a)^
Relative wall thickness	0.09 [−0.15, 0.32] ^(a)^	0.28 [0.04, 0.48] ^(a),^*	0.20 [−0.04, 0.41] ^(a)^
TR velocity, m/s	0.25 [0.02, 0.46] ^(a)^	−0.17 [0.40, 0.06] ^(a)^	0.25 [0.02, 0.46] ^(a),^*
PASP, mmHg	0.26 [0.03, 0.47] ^(a),*^	−0.16 [−0.38, 0.08] ^(a)^	0.27 [0.03, 0.48] ^(a),^*

^(a)^ Pearson (r), ^(b)^ Spearman (ρ)’s correlation coefficient, 95% CI = 95% Confidence Interval; * statistical significance: 95% CI did not contain 0; LVEDV = left ventricular end-diastolic volume, LVESV = left ventricular end-systolic volume. LVEDP = left ventricular end-diastolic pressure, LA = left atrial, IVST interventricular septum thickness, PWT = posterior wall thickness, LV = left ventricular, LVDd = left ventricular end-diastolic dimension, LVSd = left ventricular end-systolic dimension, TDE = deceleration time, TR velocity = tricuspid regurgitation peak velocity, PASP = pulmonary artery systolic pressure.

## Data Availability

The raw data involved in this study can be obtained upon reasonable request addressed to Lucia M. Procopciuc (luciamariaprocopciuc@yahoo.com).
